# Characterization of Carbapenem-Resistant *Enterobacteriaceae* with High Rate of Autochthonous Transmission in the Arabian Peninsula

**DOI:** 10.1371/journal.pone.0131372

**Published:** 2015-06-25

**Authors:** Ágnes Sonnevend, Akela A. Ghazawi, Rayhan Hashmey, Wafaa Jamal, Vincent O. Rotimi, Atef M. Shibl, Amina Al-Jardani, Seif S. Al-Abri, Waheed U. Z. Tariq, Stefan Weber, Tibor Pál

**Affiliations:** 1 Department of Medical Microbiology and Immunology, College of Medicine and Health Sciences, United Arab Emirates University, Al Ain, United Arab Emirates; 2 Department of Medicine, Tawam Hospital, Al Ain, United Arab Emirates; 3 Department of Microbiology, Faculty of Medicine, Kuwait University, Kuwait City, Kuwait; 4 Department of Pharmaceutics, College of Pharmacy, King Saud University, Riyadh, Kingdom of Saudi Arabia; 5 Department of Pathology and Pharmacology, College of Medicine, Alfaisal University, Riyadh, Kingdom of Saudi Arabia; 6 Central Public Health Laboratories, Ministry of Health, Muscat, Oman; 7 Department of Medicine, Royal Hospital, Muscat, Sultanate of Oman; 8 Department of Laboratory Medicine, Tawam Hospital, Al Ain, United Arab Emirates; 9 Department of Pathology and Laboratory Medicine, Sheikh Khalifa Medical City, Abu Dhabi, United Arab Emirates; Institut National de la Recherche Agronomique, FRANCE

## Abstract

To establish the role of local transmission versus possible pathogen import due to previous foreign exposure in infections caused by carbapenem non-susceptible *Enterobacteriaceae* in the Arabian Peninsula, 200 independent isolates collected in 16 hospitals of Saudi Arabia, Kuwait, Oman and the United Arab Emirates were studied. All strains were multidrug resistant; 42.5% of them also qualified as extremely drug resistant. The frequency of various carbapenemases varied according to the participating countries, but in the collection, as a whole, *bla*_NDM-1_ was the most frequently encountered carbapenemase gene (46.5%) followed by *bla*_OXA-48-like_ gene (32.5%). A comparatively high rate (8.9%) of multi-clonal strains carrying both *bla*_NDM_ and *bla*_OXA-48-like_ genes in the United Arab Emirates, representing the most resistant subgroup, was encountered. No KPC-expressing isolates were detected. Three major clones of *bla*_NDM-1_ carrying *Klebsiella pneumoniae* of ST152 (n = 22, Saudi Arabia), ST14 (n = 7, United Arab Emirates) and ST147 types (n = 9, Oman) were identified, the latter two clones carrying similar, but not identical HI1b incompatibility type plasmids of >170kb. While from 78.6% of the cases with documented foreign hospitalization *bla*_NDM_ positive strains were isolated, these strains formed only 25.6% of all the isolates expressing this enzyme. In fact, 56.8% of the NDM, 75.7% of OXA-48-like and 90.9% of VIM positive strains were recovered from patients without documented foreign exposure, neither in the form of travel or prior hospitalization abroad, suggesting a high rate of autochthonous infections. This, considering the extensive links of these countries to the rest of the world, predicts that trends in the local epidemiology of carbapenem resistant strains may increasingly affect the spread of these pathogens on the global scale. These results call for improved surveillance of carbapenem resistant *Enterobacteriaceae* in the countries of the Arabian Peninsula.

## Introduction

Countries of the Arabian Peninsula have rapidly developing links to the rest of the World as becoming increasingly busy business and medical tourism hubs and some of them popular holiday destinations, as well. A unique feature of their population structure is the high rate of resident expatriates (as an average, approximately 48%), which rate in Qatar and the United Arab Emirates (UAE) reaches more than 80% [1]. The majority of these expatriate workers arrive from the Indian Subcontinent, the Far East, and Africa and from other Middle Eastern countries. They, as well as citizens of the Gulf countries, frequently seek health care abroad in North America, Europe, and in their own home countries, i.e. regions frequently burdened by high rate of antibiotic resistance.

These characteristics make countries of the Arabian Peninsula highly exposed to the spread of various infectious agents, including carbapenem resistant *Enterobacteriaceae* (CRE). Indeed, these strains have been reported from sporadic cases and from small outbreaks in almost all countries of the region [2–18]. Conversely, the extensive international connections are also likely to facilitate the spread of CRE from these countries to other parts of the Globe. This role has been well documented for the broader Middle Eastern region, particularly in case of strains producing OXA-48-like and NDM type carbapenemases [19–21]. Based on a limited number documented cases in Europe and in the USA with links specifically to some of the respective countries, or to their immediate vicinity [19, 22–26], a similar role has been proposed for the Arabian Peninsula, as well [20]. Nonetheless, local information based on systematic antibiotic resistance surveillance is sparse [27]. Even less known are the similarities and differences in the epidemiology of CRE in the different countries of the Peninsula. We are aware of one study, only, with the aim to compare isolates collected from multiple countries of the region, but even in this project over 80% of the carbapenemase producing strains among the total of 62 CRE isolates studied were isolated in a single Saudi hospital [17].

From the extensive exposure of these countries one may predict that a considerable part of local CRE cases could actually be imported. However, there are no local surveillance data either to support or to oppose such predictions. A limited number of CRE infections with no previous foreign exposure were indeed documented in the region [4, 5, 7, 9, 11, 12, 28, 29]. However the actual rate of such events, suggestive of autochthonous transmission, compared to those with proven prior foreign exposure is not known. Such information is important to understand the local epidemiology of CRE and to establish local screening strategies for these pathogens when admitting patients to hospitals. Here we present our data on the distribution of various types of carbapenemases, on the antibiotic susceptibility, genotype, clustering, and travel-, and prior hospitalization-related nature of 200 independent, carbapenem non-susceptible *Enterobacteriaceae* isolates recovered in four countries of the Arabian Peninsula.

## Materials and Methods

### Strains and data collection

Altogether 265 non-repeat *Enterobacteriaceae* strains isolated between April 2009 and April 2013 from inpatients of 16 hospitals of the Kingdom of Saudi Arabia, (KSA), Kuwait, Oman and the UAE were submitted to the Department of Medical Microbiology and Immunology, UAE University. For comparison, this pool of strains included 6 *Klebsiella pneumoniae* (KKP1, 2, 4, 6, 8, 11), 1 *Escherichia coli* (KEC7) and 1 *Enterobacter cloacae* (KECL3) expressing VIM-4, and 2 NDM positive *K*. *pneumoniae* (KKP5 and 9) from Kuwait, and 6 NDM positive (strains No.1, 2, 4, 6, 13, 15), 1 OXA-48-like-positive *K*. *pneumoniae* (strain No.11) and 1 OXA-48-like-positive *E*. *coli* (strain No.3) strains from Oman that had been parts of collections studied earlier [4, 9]. All strains were isolated by clinical or epidemiological indications, and not for the purpose of the current study. The only initial criteria for an *Enterobacteriaceae* isolate to qualify for submission was that at the time of its isolation the strain exhibited decreased susceptibility to any of the carbapenems, by any methods used by the respective laboratories. After re-testing at UAE University (see later), strains with confirmed non-susceptibility by the CLSI criteria (30) to at least one of the carbapenems used (i.e. imipenem, meropenem, ertapenem) were finally included in the study. Strains were stored in duplicates at -80°C in Tryptic Soy broth (MAST Group Ltd., UK) containing 20% glycerol.

Data on age, gender, sample type, and the clinical relevance of the isolate, as assessed by the providing laboratories, along with history of travel and hospitalization within one-year prior the isolation of the strains were obtained by local participants of the project from databases complementing the strain collections of the respective laboratories. The absence of relevant data was specifically noted. For the KSA isolates, only the fact of previous hospitalization, but not its actual location was known, hence these cases were not used in the respective analyses. Access to records on nationality, clinical diagnosis, treatment and clinical outcome were limited, hence the effect of these factors was not addressed within the current study.

### Phenotypic tests

Susceptibility to ampicillin, amoxicillin-clavulanic acid, cefoxitin, piperacillin-tazobactam, cefpodoxime, ciprofloxacin, gentamicin, tobramycin, amikacin, chloramphenicol, doxycycline and trimethoprim-sulfamethoxazole was tested by disc diffusion. The Minimum Inhibitory Concentration (MIC) to ceftazidime, cefotaxime, aztreonam, imipenem, meropenem, ertapenem was determined by microdilution according to CLSI [30] using *Escherichia coli* ATCC25922 as control. Tigecycline and colistin sensitivity was assessed by Etest (bioMérieux, US) applying the EUCAST guidelines [31]. To compare susceptibilities of various groups of strains, the MIC_90_ and MIC_50_ values were used. Strains were considered as multi-, or extensively drug resistant (MDR and XDR, respectively) according to the recent recommendations [32]. The carbapenemase production of the strains was assessed by the CarbaNP test [33].

### Molecular methods

The presence of the *bla*_NDM_, *bla*_OXA-48-like,_ *bla*_VIM_, *bla*_IMP_, *bla*_KPC_ carbapenemase genes, and *armA*, *rmtA*, *B*, *C* and *D* ribosomal methylase genes were tested by PCR [7, 34]. Sequences of the entire *bla*_NDM_ [7] and *bla*_VIM_ [8] genes, and that of a 708 bp internal part of the *bla*_OXA48-like_ gene characteristic to the most common alleles [35, 36] were determined. Plasmids carrying carbapenemase genes were conjugally transferred into a Na-azide resistant derivative of *E*. *coli* J53 and their molecular mass, incompatibility type and RFLP was determined, as previously described [7]

Macrorestriction patterns of *Xba*I-digested genomic DNA obtained by pulsed field gel electrophoresis (PFGE) were compared using the GelCompar II software (Applied Maths, Belgium) [7]. A clone was tentatively defined as a PFGE cluster of the same species isolates with **≥** 80% similarity, expressing the same carbapenemase and harboring carbapenemase gene-carrying plasmids of the same molecular mass and, if typable, of the same incompatibility group. The multi-locus sequence type (MLST) of selected *K*. *pneumoniae* strains was also determined [37].

### Statistical analysis

Fisher's exact test (two tailed) was used to compare variables of different groups of strains.

### Ethics statement

The study did not make use of human or vertebrate animal subjects or tissues. The bacterial isolates studied were obtained from existing strain collections routinely assembled as part of clinical laboratory practices saving multi-drug resistant organisms and no specimens were collected for the purpose of this study. Strains studied were accompanied by data only that did not allow any patient identification (i.e. no names, birth dates, personal-, hospital-, or laboratory identification numbers were known) and the data were analyzed anonymously. Collection of the strains in each of the participating clinical laboratories was conducted in accordance with the Declaration of Helsinki and with the particular institutional ethical and professional standards.

## Results

### The collection

After confirming their carbapenem non-susceptibility, altogether 200 non-repeat strains were included in the study. One hundred and seventy of the isolates (85.0%) were judged clinically relevant by the clinical microbiologists of the submitting laboratories. The details of the strains, patients, specimens and the list of providing hospitals according to the participating countries are shown in Table 1.

**Table 1:** Distribution of the isolates, patients, specimen types and hospitals among the participating countries.

		All	KSA		Kuwait		Oman		UAE	
**The isolates (N)**	All	200	54		27		63		56	
	*K*. *pneumoniae*	145	46		17		41		41	
	*E*. *coli*	28	3		5		10		10	
	*E*. *cloacae*	17	4		3		7		3	
	Others	10	1		2		5		2	
**The patients**	Age in years (x±sd)	53.2 ± 22.0	57.1 ± 12.9		54.7 ± 27.5		47.9 ±24.8		55.3 ± 22.6	
	Male:female ratio	1.8	3.15		0.92		1.42		2.16	
**The specimens (%)**	Respiratory	29.5	70.4		0.0		11.1		25.0	
	Blood	15.5	20.4		14.8		9.5		17.9	
	Pus	19.0	1.9		25.9		39.7		8.9	
	Urine	21.0	7.4		59.3		19.0		17.9	
	Screening	11.5	0.0		0.0		20.6		17.9	
	Unknown	3.5	0.0		0.0		0.0		12.5	
**The hospitals (N of isolates)**			A	(22)	Mubarak	(16)	Royal	(63)	Mafraq	(17)
			B	(15)	Adan	(5)			Tawam	(17)
			C	(17)	Amiri	(3)			SKMC	(4)
					Ibn Sina	(3)			Zayed	(2)
									Al Ain	(1)
									Quasimi	(12)
									Kuwait	(2)
									Dubai City	(1)

Beyond *K*. *pneumoniae* (72.5%), *E*. *coli* (14.0%) and *E*. *cloacae* (8.5%). *Citrobacter freundii* was represented by 3, *Morganella morganii* and *K*. *oxytoca* by 2, and *C*. *koseri*, *Serratia marscescens* and *Providencia stuartii* by 1 isolates, each, respectively.

### Distribution of carbapenemase and other antibiotic resistance genes

The most common single carbapenemase gene encountered was *bla*_NDM_ carried by 93 (46.5%) of the isolates followed by *bla*_OXA-48-like_ and *bla*_VIM_ (Table 2). Neither *bla*_IMP_, nor *bla*_KPC_ carriers were detected. The 23 isolates (11.5%), in which none of the five carbapenemase genes targeted were found, did not exhibit any carbapenemase activity. There were notable differences between countries regarding the frequencies of carbapenemase genes. While in the Saudi, Omani and in the Emirati collections the most common carbapenemase gene detected was the *bla*_NDM_ followed by the *bla*_OXA-48-like_ gene, in the Kuwaiti collection *bla*_VIM_ was the most frequently encountered one. However, once strains previously reported from this country [9] were excluded, none of the remaining 17 Kuwaiti isolates carried this latter gene, and among them the *bla*_OXA-48-like_ gene (41.2%) was the most frequent one, followed by non-carbapenemase producing isolates (35.2%) and by *bla*_NDM_ carrying ones (23.5%) (*data not shown*).

**Table 2:** Frequencies of different resistance genes among the isolates.

Resistance classes	Resistance gene	Entire collection (%*) (N = 200)	Local collections (%^&^)			
			KSA (N = 54)	Kuwait (N = 27)	Oman (N = 63)	UAE (N = 56)
**Carbapenemases**	*bla*_NDM_	46.5	57.4	22.2	46.0	48.2
	*bla*_OXA48-like_	32.5	33.3	25.9	44.4	21.4
	*bla*_NDM_ + *bla*_OXA48-like_	3.5	1.9	0.0	1.6	8.9
	*bla*_VIM_	6.0	1.9	29.6	3.2	1.8
	No carbapenemase gene	11.5	5.6	22.2	4.8	19.6
**Ribosome-modifying enzymes**	*armA*	15.5	11.1	0.0	23.8	17.9
	*rmtB*	0.5	0.0	0.0	1.6	0.0
	*rmtC*	21.5	57.4	14.8	11.1	1.8

* % calculated in the entire collection

^&^ % calculated in the respective collections of specific countries

All *bla*_NDM_ genes detected represented the *bla*_NDM-1_ allelic variant with the exception of two *E*. *coli* strains (Oman and UAE) carrying the *bla*_NDM-7_ allele. Apart from one Emirati *K*. *pneumoniae* strain carrying *bla*_OXA-162,_ the sequences of a 708 bp long internal parts of the *bla*_OXA-48-like_ genes were consistent with that of the *bla*_OXA-48_ allele in all strains producing OXA-type carbapenemase, only. On the other hand, the *bla*_OXA_ genes of the 7 *K*. *pneumoniae* strains also carrying the *bla*_NDM_ gene exhibited a much higher variability. Their respective 708 bp internal sequences were suggestive of *bla*_OXA-48_ (in 1 KSA and 1 UAE isolates), of *bla*_OXA-162_ (1 strain form the UAE), of *bla*_OXA-181_ (1 Omani strain) and *bla*_OXA-232_ in 3 isolates from the UAE, respectively. Similarly to the Kuwaiti strains present in the collection and described earlier [9] all new VIM-positive isolates expressed the VIM-4 allele.

64.5% of all strains with *bla*_NDM_, as a single carbapenemase gene, also carried a 16S modifying enzyme gene responsible for aminoglycoside resistance, significantly more frequently (P<0.0001) than those expressing OXA-48-like carbapenemases (13.8%).

The distribution of genes coding for carbapenemases in the three most common species in general, as well as by sample types is shown in Table 3. Altogether, NDM positive *K*. *pneumoniae* was the largest sub-group (39.0%) in the entire collection. In the mixed species group, the *bla*_OXA-48-like_ gene was present in 2 *K*. *oxytoca*, and in 1 *C*. *freundii*, *C*. *koseri* and *S*. *marcescens* strains, while 2 *C*. *freundii* and 2 *M*. *morganii* strains were *bla*_NDM_ positive. One *P*. *stuartii* strain did not carry any of the carbapenemase genes targeted.

**Table 3:** Distribution of carbapenem resistance mechanisms in isolates recovered from various specimen.

Species	Carbapenemase produced	% in						
		All specimens	Respiratory	Blood	Urine	Pus	Unknown	Screening
All (N = 200)	NDM	46.5	54.2	38.7	42.9	47.4	14.3	52.2
	OXA	32.5	33.9	29.0	31.0	39.5	14.3	30.4
	NDM-OXA	3.5	3.4	3.2	4.8	2.6	0.0	4.3
	VIM	6.0	1.7	12.9	9.5	7.9	0.0	0.0
	none	11.5	6.8	16.1	11.9	2.6	71.4	13.0
*K*. *pneumoniae* (N = 145)	NDM	53.8	53.8	52.4	48.1	60.0	25.0	62.5
	OXA	29.7	36.5	9.5	37.0	28.0	25.0	25.0
	NDM-OXA	4.8	3.8	4.8	7.4	4.0	0.0	6.3
	VIM	4.1	0.0	14.3	7.4	4.0	0.0	0.0
	none	7.6	5.8	19.0	0.0	4.0	50.0	6.3
*E*. *coli* (N = 28)	NDM	32.1	66.7	20.0	42.9	25.0	0.0	0.0
	OXA	32.1	16.7	60.0	14.3	75.0	0.0	33.3
	NDM-OXA	0.0	-	-	-	-	-	-
	VIM	3.6	0.0	0.0	14.3	0.0	0.0	0.0
	none	32.1	16.7	20	28.6	0.0	100.0	66.7
*E*. *cloacae* (N = 17)	NDM	11.8	0.0	0.0	0.0	28.6	0.0	0.0
	OXA	47.1	0.0	75.0	25.0	42.9	0.0	100.0
	NDM-OXA	0.0	-	-	-	-	-	-
	VIM	29.4	100.0	0.0	25.0	28.6	0.0	0.0
	none	11.8	0.0	25.0	50.0	0.0	0.0	0.0

*bla*_OXA-48-like_ positive *K*. *pneumoniae* were recovered from blood samples significantly less frequently than other species expressing the same enzyme (P = 0.0043), or the same species with other carbapenemases (P = 0.0349). No further, statistically significant correlation between the species or resistance mechanism and the site of infection could be established.

### Antibiotic susceptibility

All isolates scored at least of multi-drug resistant (MDR) [32], while 42.5% of them also fulfilled the criteria for extremely drug resistant (XDR) with rates considerably varying between the countries (Table 4). Strains carrying both *bla*_NDM_ and *bla*_OXA-48-like_ genes were the most, and those expressing OXA-48-like carbapenemase alone were the least likely to be XDR. Only six strains in the entire collection exhibited decreased susceptibility to both tigecycline and colistin, representing all participating countries at least by one isolate. Beyond these two agents, amikacin and chloramphenicol were the most active antibiotics with 50.5% and 54.0% non-susceptibility rates, respectively (data not shown).

**Table 4:** Antibiotic susceptibility of the strains by country, by resistance mechanism and by species.

	ERT		IMI		MER		CAZ		TIG			COL*			XDR
	MIC_50_	MIC_90_	MIC_50_	MIC_90_	MIC_50_	MIC_90_	MIC_50_	MIC_90_	MIC_50_	MIC_90_	R (%)	MIC_50_	MIC_90_	R (%)	%
All	16	>64	8	32	32	128	>128	>128	1	4	22	0.19	0.38	4.1	42.5
Country															
KSA	16	64	16	32	32	64	>128	>128	1	2	7.4	0.19	0.38	7.5	18.5
Kuwait	16	64	8	128	16	128	>128	>128	1	4	25.9	0.25	0.38	4.0	33.3
Oman	16	64	8	16	16	64	>128	>128	1	4	27.0	0.125	0.38	3.2	46.0
UAE	32	>64	8	32	64	128	>128	>128	1	4	28.6	0.19	0.25	1.8	66.0
Carbapenemase															
NDM	16	>64	16	32	32	>128	>128	>128	1	4	22.6	0.19	0.38	4.4	49.4
OXA-48-like	8	32	8	32	16	32	128	>128	1	4	16.9	0.19	0.38	3.1	29.2
NDM+OXA	>64	>64	16	128	128	>128	>128	>128	2	4	50.0	0.19	0.25	0.0	71.4
VIM	8	32	8	32	16	128	128	>128	1.5	5	33.3	0.125	0.25	8.3	58.3
None	64	>64	4	8	64	128	>128	>128	0.75	4	21.7	0.19	0.38	9.1	34.8
Species															
*K*. *pneumoniae*	16	>64	8	32	32	>128	>128	>128	1.5	4	26.2	0.19	0.38	4.8	46.2
*E*. *coli*	16	>64	8	16	16	>128	>128	>128	0.38	1	0.0	0.19	0.38	3.6	21.4
*E*. *cloacae*	8	32	8	16	16	32	128	>128	1	3	17.6	0.19	0.25	0.0	41.2

ERT-ertapenem, IMI—imipenem, MER—meropenem, CAZ—ceftazidime, TIG—Tigecycline, COL—colistin, XDR—extensively drug resistant

* without *Proteus* and *Morganella*

### Clonality of the isolates

Of all the *K*. *pneumoniae*, *E*. *coli* and *E*. *cloacae* strains subjected to genomic restriction digestion 5 strains (3 NDM-expressing *E*. *coli*, 1 *K*. *pneumoniae* carrying the *bla*_OXA48-like_ gene, and 1 without carbapenemases, respectively) were non-typable. With the exception of two duplets among *E*. *coli* and two triplets among *E*. *cloacae* no clustering was observed among strains of these two species. Among the 143 *K*. *pneumoniae* strains typed, 6 PFGE clusters (1–6) (1 with 4, 2 with 6, 1 with 9, 1 with 10 and 1 with 23 members) were identified (S1–S3 Figs). After excluding clusters 2 and 4 due to the variety of carbapenemases expressed, and cluster 1 due the variation in plasmid profiles and exhibiting 4 different sizes of pNDMs (data not shown), 3 tentative clones (A-C, Table 5) were identified. All clones expressed *bla*_NDM-1_ (Table 5). The Omani clone C (n = 9) contained four members previously described (strains No.1, 2, 6 and 15 in [4]) beyond 5 newly encountered ones. Members of the 3 clones together represented 26.2% of all, and 48.7% of the *bla*_NDM-1_ positive, typable *K*. *pneumoniae* strains.

**Table 5:** Characteristics of the major clones encountered.

					NDM plasmid		
Clones	Country	*n*	Hospital (*n* of isolates)	MLST	Mass (kb)	Inc	Genes co-transferred with *bla*_NDM_
A	KSA	*22*	Hospital A (*12*) Hospital B (*6*) Hospital C (*4*)	ST152	110	NT	*rmtC*
B	UAE	*7*	Kuwait Hospital (*2*) Qasimi Hospital (*5*)	ST14	>170	HI1b	*bla*_CTX-M_ **or** armA*
C	Oman	*9*	Royal Hospital (*9*)	ST147	>170	HI1b	*bla*_CTX-M_ **and** *armA*

NT = nontypable

* in one strain the *ctxM*, in the other one the *armA* gene co-transferred with the NDM plasmid

The molecular mass and incompatibility type (HI1b) of the NDM plasmids in the Emirati clone B and in the Omani clone C, as assessed after conjugally transferring them to a suitable recipient (S1 Table) were similar (i.e. >170kb) (Table 5). While plasmids in the two Omani isolates yielded RFLP patterns indistinguishable by the naked eye with any of the enzymes (*XbaI*, *BamHI* and *SmaI*) used, their restriction patterns were noticeably different from those of the UAE isolates, which also exhibited differences between each other (S4 Fig).

### Foreign travel and previous hospitalization

Data on foreign travel during the year before the strains were isolated were available from 150 patients (75.0%) and 23.3% of them had a positive travel history. Sixty per cent of the travel destinations were India, 14.3% Africa, 8.6% Pakistan, and 5.7% each of the Middle East, South East Asia or other regions, respectively. Among the patients with NDM, OXA and VIM-expressing strains positive travel history was recorded for 29.3%, 20.0% and 8.3% of the cases, respectively. One hundred and fifteen patients (72.3% of those with records available) had been treated as in-patients during the year preceding current hospitalization. Previous hospitalization had been documented among 67.5%, 78.9% and 83.3% of those from whom strains with the *bla*_NDM_, *bla*_OXA-48-like_ or *bla*_VIM_ genes were isolated, respectively.

Strains isolated from patients who had travelled or had been previously hospitalized were more likely to express the XDR phenotype than other strains. On the other hand the relation with the different carbapenemase types was more mixed (Table 6). The *bla*_NDM_ gene, as a single carbapenemase gene was more common among strains isolated from patients with a positive travel history, but less frequent among previously hospitalized individuals. The frequencies of the *bla*_OXA-48-like_ and *bla*_VIM_ carbapenemase genes showed exactly the opposite trend: they were more common among non-travellers and among previously hospitalized individuals (Table 6). Irrespective of the differences observed, none of these correlations were statistically significant. Data were not available in sufficiently high number for NDM-OXA double positive strains to carry out a similar analysis.

**Table 6:** The rate of various carbapenemases in different patient groups.

Patient groups	Frequency of (%)			
	XDR	NDM	OXA	VIM
All with travel data (N = 150)	36.7	50.0	36.5	8.0
Travelled (N = 35)	45.7	62.9	31.4	2.9
Not travelled (N = 115)	33.9	46.1	38.3	9.6
All with hospitalization data (N = 159)	38.4	50.3	35.8	7.5
Hospitalized (N = 115)	42.6	47.0	39.1	8.7
Non hospitalized (N = 44)	27.3	59.1	27.3	4.5

For 95 previously hospitalized patients from Kuwait, Oman and from the UAE the actual location of prior inpatient care was known (Table 7). 78.6% of them had previously been admitted to a hospital in India, 14.3% in Pakistan and 7.1% in Sudan, respectively. From 78.6% of these patients *bla*_*NDM*_-carrying strains were isolated, significantly more (P = 0.0087) than from those treated earlier in a domestic hospital. In contrast, OXA-expressing strains were significantly more common among former inpatients of the same hospital (P = 0.0059) than of any other hospitals. However, even with this high rate of *bla*_NDM_ positive isolates among patients with foreign hospitalization history, records of any recent inpatient care abroad was present in 25.6% of the patients with NDM positive isolates, only. The respective figure was even lower among OXA-48-like isolates (8.1%), and no foreign hospitalization was recorded for any of the cases with VIM-positive strains.

**Table 7:** Frequency of strains producing different carbapenemases and expressing the XDR phenotype among patients with different hospitalization history.

Carbapenemase/ phenotype	% in patient groups previously hospitalized in				
	Same hospital (N = 52)	Any other hospital (N = 43)	Other domestic hospital (N = 29)	All domestic hospitals (N = 81)	Foreign hospital (N = 14)
All (N = 95)*	54.7	45.3	30.5	85.3	14.7
XDR	40.4	53.5	51.7	44.4	57.1
NDM	26.9	67.4	62.1	39.5	78.6
OXA	51.9	23.3	24.1	42.0	21.4
VIM	13.5	7.0	10.3	12.3	0.0

* including NDM-OXA double positive and carbapenemase negative isolates, as well.

In 69.8% of the 96 CRE cases with all data available no foreign exposure (i.e. neither travel nor foreign hospitalization) could be documented with local figures of 73.7%, 69.8% and 64.3 in Kuwait, Oman and the UAE respectively. No foreign exposure was present in 90.9% and 75.7% of patients infected with VIM and OXA-48-like producing isolates, respectively. Even among those infected with NDM positive strains the complete lack of foreign exposure was as common as 56.8%.

## Discussion

CRE infections occurring internationally with direct links to the countries of the Arabian Peninsula have been described [21, 24–26, 38, 39] suggesting that the region can serve as source of these strains, just other "reservoir" areas identified earlier do [20]. However, despite the sporadic reports of local cases with no history of travel or foreign hospitalization [4, 5, 7, 9, 29] it has not been known at what rate local cases are likely to be acquired within the borders of the countries of the Peninsula and how these figures relate to the different type of carbapenemases commonly found in the region.

Our results, based on the largest number of strains studied in the region so far, confirmed previous observations [4, 5, 7, 9, 13–15, 28] that currently NDM and OXA-48-like enzymes are the two major carbapenemases of *Enterobacteriaceae* present in most countries of the Peninsula, albeit with regionally varying proportions (Table 2). The fact that among Kuwaiti isolates *bla*_VIM-4_ was the most commonly encountered one was clearly due to the fact that, for comparison, we included strains from a previous study describing a high incidence of VIM-4 in that country [9]. Disregarding this latter subgroup of isolates no further strain expressing this carbapenemase was found and the frequencies of OXA-48-like and NDM enzymes were comparable to those seen in the other countries. It should be noted that while *bla*_VIM-4_-carrying strains have also been encountered earlier in the UAE [8] and in the KSA [13, 14], this is the first report on the presence of this carbapenemase type in Oman. NDM-OXA double positive strains, whith their comparatively high rate (8.9%) among the UAE isolates have been noted earlier in some of these countries [4, 17, 28]. The fact that in our study these were the isolates most likely to be XDR (Table 4) worth attention.

Regarding the allelic types of the different carbapenemases encountered it was noteworthy that the variant *bla*_NDM-7_, detected in two unrelated *E*. *coli* strains, (one from the UAE and one from Oman) had already been reported in connection to the Peninsula, i.e. in a strain isolated from a Yemeni patient in Germany [39]. Based on the determination of an internal sequence characteristic to the most common OXA-48-like alleles [36], one of our Omani isolates carried a gene consistent with the respective sequence of the *bla*_OXA-181_ variant, i.e. an allele repeatedly described earlier in the same country [4, 6]. As far as we are aware of, neither the *bla*_OXA-162_ nor the *bla*_OXA-232_ allele have been reported earlier from the Arabian Peninsula.

The apparent absence of KPC-producing strains in the Arabian Peninsula is a particularly interesting observation. While outbreaks have been documented in other parts of the Middle East [40], from countries of the Peninsula this carbapenemase had only been reported once before in an *E*. *coli* ST131 strain [41], and recently by us in two *K*. *pneumoniae* isolates from the UAE [29]. Further studies should reveal whether the apparent local rarity of this carbapenemase, otherwise broadly spread globally, is a temporary, incidental phenomenon, only or it reflects yet to identified specifics of local antibiotic usage or other factors of public health. Similarly, further invastigations are needed to clarify whether the notably higher rate of XDR strains (66.0%) in the UAE (Table 4) is a finding biased by the relatively small number of isolates studied or it is a result of differences in local therapy guidelines and practices. Regretfully, such data were not available to us to compare them with the respective susceptibility figures.

It was also noteworthy, that while having the lowest rate of XDR strains (Table 4), the highest frequency of colistin resistance was found in the KSA collection, a phenomenon well known in that country [42]. Importantly, this was due to sporadic isolates, as only one strain of the 22-member sized cluster A of Saudi isolates exhibited resistance to colistin *(data not shown)*.

The frequent association of 16S methylase-coding genes with plasmids coding for carbapenemases, particularly for *bla*_NDM_, has been known [43, 44] and our data support that observation. Their wide-spread nature and their highly significant association of these genes with the *bla*_NDM_-carrying plasmids are important factors in limiting the therapeutic options for these infections.

We are not aware of previous reports from this region on strains similar to those of the Saudi clone A of the current study (Table 5). On the other hand, an ST14 *K*. *pneumoniae* strain, similar to those in Emirati clone B carrying a >170 kb plasmid with *bla*_NDM-1_ and *armA* genes, had earlier been detected in Oman [5]. While the NDM plasmid in that isolate belonged to a different, i.e. IncL/M incompatibility group, a similar strain isolated in the UAE with a plasmid of the same, i.e. HI1b, incompatibility type was also described [7]. Furthermore, similar IncHI1b plasmids, but in ST147 type strains, had also been reported from Oman [4] and 4 of those strains, together with 6 of our new isolates, formed clone C of the current study. Although transfer of these large, conjugative, IncHI1b NDM plasmids between ST14 (clone B) and ST147 strains (clone C) would offer a plausible explanation for the similarities between episomes of clones B and C, the RFLP did not confirm complete identity of these plasmids (S4 Fig). Further, more detailed analysis of these plasmids is needed to assess the real level of differences between them.

An important finding of this study, shown for the first time, that among CRE infections in the countries of the Arabian Peninsula the lack of foreign exposure is not restricted to a limited number reported previously [4, 5, 7, 9, 11, 12, 28]. In fact, in the majority of the cases (69.8%, varying between 64.3% and 73.7% in the participating countries) we could not demonstrate any history of foreign travel or hospitalization abroad. This finding, contrary to what one may anticipate from the local demography data and from common use of health care abroad, strongly suggests that autochthonous transmission is likely to be a key factor in the epidemiology of CRE in countries of the Peninsula. Although the above statement was true for all CRE types encountered, the lack of foreign exposure was more common for *bla*_OXA-48-like_ and for *bla*_VIM_ positive, than for NDM-1 expressing strains. Actually, for strains carrying the latter gene, previous hospitalization abroad (mostly in India in this study) still seems to be a risk factor, even though the majority of such strains (56.7%) still appear to be acquired domestically. Based on these findings it is questionable whether local pre-admission screening criteria can include previous foreign hospitalization/exposure as recommended in several Western countries.

There are several limitations of the current study. Due to criteria by which the current collection of strains was compiled and without the respective data on carbapenem susceptible isolates available, our results does not tell anything about the incidence or prevalence of CRE in the participating countries. Another limitation is that patients had not been screened at admission, or those results were not available to us. i.e. we do not know whether patients were admitted as carriers or had acquired the strains by nosocomial transmission. Nevertheless, in case of NDM positive isolates, based on the statistically significant correlation, which are in line with previous observations regarding the Indian subcontinent as a frequent source [20, 22, 24, 43], and also on the completely opposite trend seen with OXA-48-like expressing strains, importing NDM-1 positive isolates, mostly from India, is still likely to be an important, albeit not the main contributing factor to the prevalence of these strains in the Arabian Peninsula. The findings also highlight the need for the yet to be developed data on the rate of symptomless carriers of CRE in these communities. The finding that in this study 14.0% of all CRE were *E*. *coli* is of high relevance, as representatives of this species are more likely to colonize the gut efficiently and spread the problem outside of the hospitals.

Taken together, the high rate of local transmission suggested by our data, along with the extensive and fast-developing links of these countries to the rest of the world predict that in the future local trends may increasingly impact the global epidemiology of CRE. Only extensive, improved national surveillance strategies combined with close cooperation between countries of the region and beyond carry the promise to contain this fast emerging problem globally and in the countries of the Arabian Peninsula.

## Supporting Information

S1 Fig PFGE dendogram of *K*. *pneumoniae* strains. PFGE clusters (>3 members) are numbered (1–6) and boxed. PFGE clusters marked by dashed lines were not considered “clones” as either not having more than 3 members expressing the same carbapenemases (clusters 2 and 4) or exhibiting variable plasmid profiles and having the same carbapenemase gene located of plasmids of different sizes (cluster 4). Clusters marked by continuous lines were considered clones A-C. The 80% similarity threshold is marked by a horizontal dotted line. * Indicates strains from [9] and ** marks isolates from [4].
(TIFF)

S2 Fig PFGE dendogram of *E*. *coli* strains. The 80% similarity threshold is marked by a horizontal dotted line. * Indicates strains from [9] and ** marks isolates from [4].
(TIFF)

S3 Fig PFGE dendogram of *E*. *cloacae* strains. The 80% similarity threshold is marked by a horizontal dotted line. * Indicates strains from [9].
(TIFF)

S4 Fig Restriction fragment length polymorphism patterns of the plasmids from the Omani and Emirati clones. M: Lamda *HindIII* digest molecular mass standard. A and B: UAE strains ABC119 and ABC130. C and D: Omani strains OM34 and No.2. Panel 1:*XbaI*; Panel 2: *BamHI*, Panel 3: *SmaI* digests.
(TIFF)

S1 Table Antibiotic susceptibility of pNDM transconjugants. & Minimal inhibitory concentration, microdilution method. ¶ Disc diffusion. ETP—ertapenem, MEM—meropenem, IMI—imipenem, CAZ—ceftazidime, CTX—cefotaxime, AZT—aztreonam, CIP—ciprofloxacin, GM—gentamicin, AM—amikacin, TO—tobramycin, CHL—chloramphenicole, DOX—doxycycline, TMP-SMX—trimethoprime-sulphamethoxazole, WT—wild type, TC—transconjugant, R–Recipient. * strain identical to isolate No.2 in [4].
(DOCX)
