# Supplementary figures and images for: Characterization of Carbapenem-Resistant Enterobacteriaceae with High Rate of Autochthonous Transmission in the Arabian Peninsula
Source: PLoS One. 2015 Jun 25;10(6):e0131372. doi: 10.1371/journal.pone.0131372 (PMC4482506; doi:10.1371/journal.pone.0131372)

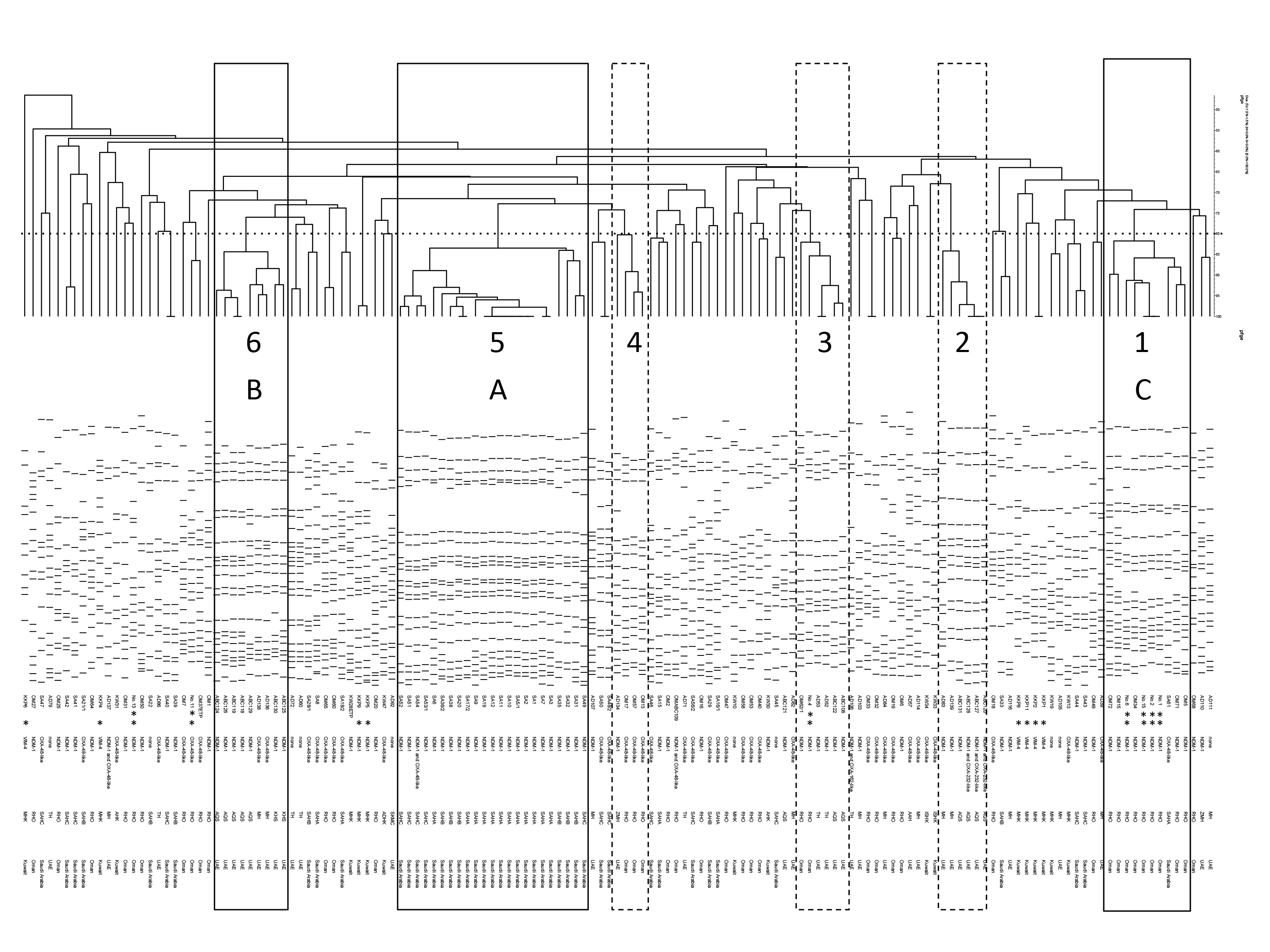

Supplement: S1 Fig — PFGE clusters (>3 members) are numbered (1–6) and boxed. PFGE clusters marked by dashed lines were not considered “clones” as either not having more than 3 members expressing the same carbapenemases (clusters 2 and 4) or exhibiting variable plasmid profiles and having the same carbapenemase gene located of plasmids of different sizes (cluster 4). Clusters marked by continuous lines were considered clones A-C. The 80% similarity threshold is marked by a horizontal dotted line. * Indicates strains from [9] and ** marks isolates from [4]. (TIFF) [file pone.0131372.s001.tiff]

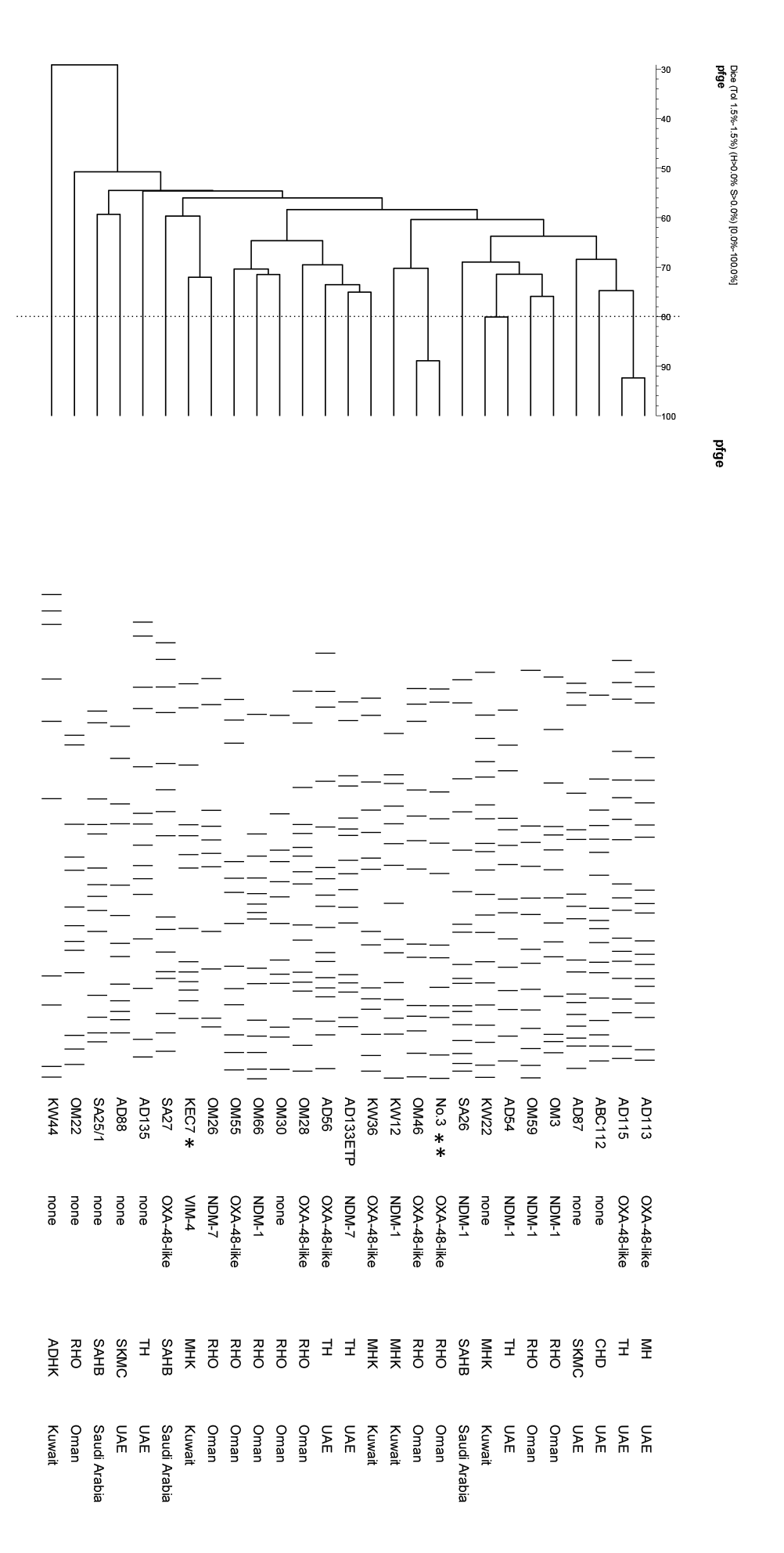

Supplement: S2 Fig — The 80% similarity threshold is marked by a horizontal dotted line. * Indicates strains from [9] and ** marks isolates from [4]. (TIFF) [file pone.0131372.s002.tiff]

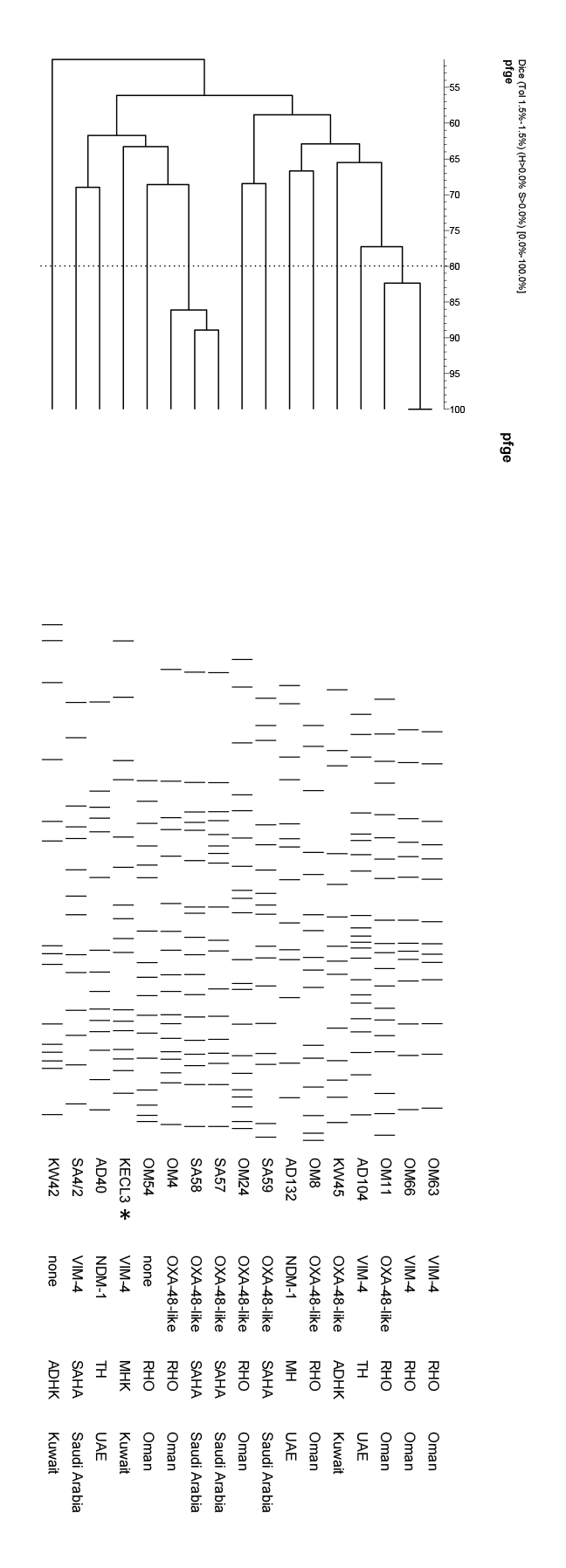

Supplement: S3 Fig — The 80% similarity threshold is marked by a horizontal dotted line. * Indicates strains from [9]. (TIFF) [file pone.0131372.s003.tiff]

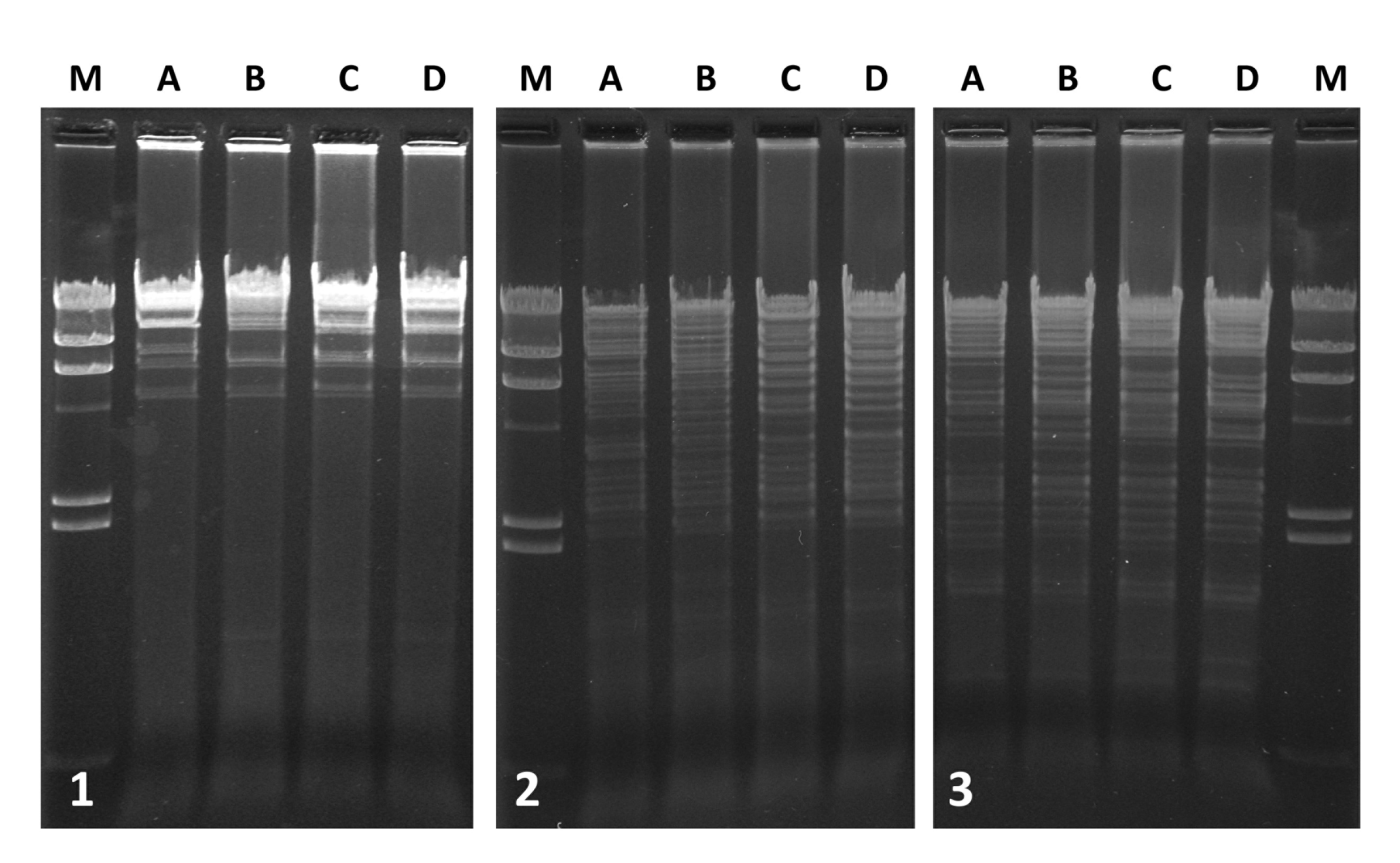

Supplement: S4 Fig — M: Lamda HindIII digest molecular mass standard. A and B: UAE strains ABC119 and ABC130. C and D: Omani strains OM34 and No.2. Panel 1:XbaI; Panel 2: BamHI, Panel 3: SmaI digests. (TIFF) [file pone.0131372.s004.tiff]
