# Supplementary material for: Characterization of Carbapenem-Resistant Enterobacteriaceae with High Rate of Autochthonous Transmission in the Arabian Peninsula
Source: PLoS One. 2015 Jun 25;10(6):e0131372. doi: 10.1371/journal.pone.0131372 (PMC4482506; doi:10.1371/journal.pone.0131372)
Supplement: S1 Table — & Minimal inhibitory concentration, microdilution method. ¶ Disc diffusion. ETP—ertapenem, MEM—meropenem, IMI—imipenem, CAZ—ceftazidime, CTX—cefotaxime, AZT—aztreonam, CIP—ciprofloxacin, GM—gentamicin, AM—amikacin, TO—tobramycin, CHL—chloramphenicole, DOX—doxycycline, TMP-SMX—trimethoprime-sulphamethoxazole, WT—wild type, TC—transconjugant, R–Recipient. * strain identical to isolate No.2 in [4]. (DOCX) [file pone.0131372.s005.docx]

**S1 Table** Antibiotic susceptibility of pNDM transconjugants

| Strain | Strain type | MIC^&^ (mg/L) | | | | | | Diameter of zone of inhibition^¶^ (mm) | | | | | |
| --- | --- | --- | --- | --- | --- | --- | --- | --- | --- | --- | --- | --- | --- |
|  |  | ETP | MEM | IMI | CAZ | CTX | AZT | CIP | GM | AM | TO | DOX | TMP-SMX |
| SA7 | WT | 32 | >128 | 16 | >128 | >128 | 128 | 0 | 0 | 0 | 0 | 12 | 0 |
| J53RAZ(pSA7/6) | TC | 0.5 | 16 | 2 | >128 | 32 | <0.25 | 32 | 0 | 0 | 0 | 25 | 35 |
| SA20 | WT | 32 | >128 | 16 | >128 | >128 | 128 | 0 | 8 | 0 | 0 | 22 | 0 |
| J53RAZ(pSA20/8) | TC | 0.5 | 16 | 4 | >128 | 16 | <0.25 | 33 | 0 | 0 | 0 | 24 | 37 |
| ABC119 | WT | >64 | >128 | 64 | >128 | >128 | >128 | 0 | 0 | 0 | 0 | 13 | 0 |
| J53RAZ(pABC119/2) | TC | 0.5 | 4 | 2 | >128 | 32 | <0.25 | 28 | 0 | 0 | 0 | 26 | 31 |
| ABC130 | WT | 32-16 | 64 | 1 | >128 | >128 | >128 | 0 | 7 | 19 | 8 | 9 | 0 |
| J53RAZ(pABC130/7) | TC | 0.5 | 4 | 1 | >128 | >128 | 4 | 29 | 26 | 26 | 16 | 25 | 34 |
| OM34 | WT | 16 | 32 | 16 | >128 | >128 | >128 | 0 | 0 | 0 | 0 | 0 | 0 |
| POM34/6 | TC | 0.25 | 4 | 1 | >128 | >128 | 4 | 29 | 0 | 0 | 0 | 25 | 32 |
| OM39* | WT | 32 | 64 | 16 | >128 | >128 | >128 | 0 | 0 | 0 | 0 | 0 | 0 |
| POM39/5 | TC | 0.25 | 8 | 1 | >128 | >128 | 4 | 32 | 0 | 0 | 0 | 25 | 32 |
| J53RAZ | R | <0.125 | <0.25 | <0.25 | <0.25 | <0.25 | <0.25 | 32 | 22 | 28 | 22 | 26 | 36 |

& Minimal inhibitory concentration, microdilution method

¶ Disc diffusion

ETP - ertapenem, MEM - meropenem, IMI - imipenem, CAZ - ceftazidime, CTX - cefotaxime, AZT - aztreonam, CIP - ciprofloxacin, GM - gentamicin, AM - amikacin, TO - tobramycin, CHL - chloramphenicole, DOX - doxycycline, TMP-SMX - trimethoprime-sulphamethoxazole

WT - wild type, TC - transconjugant, R - Recipient

* strain identical to isolate No.2 in (4).
